# Changes in bacterial composition and metabolite profiles during kimchi fermentation with different garlic varieties

**DOI:** 10.1016/j.heliyon.2024.e24283

**Published:** 2024-01-09

**Authors:** Yun-Jeong Choi, Ju-Young Lim, Min-Jung Kang, Ji-Young Choi, Ji-Hee Yang, Young Bae Chung, Sung Hee Park, Sung Gi Min, Mi-Ai Lee

**Affiliations:** a Kimchi Industry Promotion Division, World Institute of Kimchi, Gwangju, 61755, Republic of Korea; b Namhae Garlic Research Institute, Namhae, 52430, Republic of Korea

**Keywords:** Kimchi, Garlic, Metabolites, Microbial community, Lactic acid bacteria

## Abstract

Garlic, a key ingredient in kimchi, is an indispensable source of lactic acid bacteria, which are essential for fermentation. This study explored the effects of various garlic varieties on kimchi fermentation, focusing on changes in microbial communities and metabolite profiles. We observed that the type of garlic used did not significantly alter the microbial community. However, the presence of garlic itself made a significant difference. Specifically, kimchi with garlic showed higher abundance of *Leuconostoc* and *Weissella*, which are bacteria primarily responsible for kimchi fermentation. Additionally, kimchi containing garlic had increased levels of mannitol and fructose, which significantly influence taste; however, lactic acid and putrescine levels were decreased. Therefore, the addition of garlic directly contributes to the flavor profile of kimchi. Sixty-two metabolites were identified using gas chromatography and mass spectrometry. The variety of garlic added influenced the metabolite profiles of kimchi, particularly in the later stages of fermentation. These profiles were categorized based on the garlic's origin, whether from southern or northern ecotypes (*R*^*2*^*X* = 0.933, *R*^*2*^*Y* = 0.986, *Q*^*2*^ = 0.878). These findings confirm that both the presence and the variety of garlic significantly impact the microbial ecology and metabolites during kimchi fermentation, underscoring its essential role in the process.

## Highlights

• Garlic addition influenced kimchi fermentation.
• *Weissella* was relatively abundant in kimchi with garlic.
• Mannitol increased in garlic-added kimchi, while putrescine decreased.
• *Leuconostoc* and *Weissella* were positively correlated with most metabolites.
• Overall, metabolite profiles in kimchi were evidently changed by garlic varieties.

## 1 Introduction

Garlic (*Allium sativum* L.), a crop belonging to the family Liliaceae, has been widely used in our diet since ancient times because of its unique flavor components. Garlic enhances the taste of food, preserves food, and inhibits the proliferation of pathogenic bacteria in food [1]. Garlic is an essential ingredient of kimchi, as it inhibits the proliferation of unwanted bacteria derived from various raw materials during kimchi fermentation [2]. Allicin, extracted from garlic, is an antibacterial substance that directly affects the dominance of kimchi lactic acid bacteria (LAB) during initial fermentation [3]. Some studies have shown that certain LAB, which produce antibacterial substances, are present in garlic [4,5]. LAB derived from kimchi cabbage and garlic is essential for kimchi fermentation [6,7]. According to a recently published study [5,7,8], garlic provides the LAB necessary for kimchi fermentation and significantly impacts the changes in the microbial community during the fermentation process. However, the effects of garlic on the microbial communities and metabolites in kimchi remain to be investigated in detail.

Kimchi is prepared by fermenting a mixture of major ingredients, such as kimchi cabbage and radishes, and sub-ingredients like garlic, green onion, ginger, and red pepper powder [9,10]. Factors that affect the taste or flavor of kimchi include volatile compounds, microorganisms, and metabolites produced by microorganisms, which vary greatly depending on the growing region of the raw material, the place where it is processed, and the fermentation conditions [[11], [12], [13]]. Besides the main ingredients and fermentation conditions, sub-components play a crucial role in influencing kimchi fermentation [14]. While some studies have explored microbial community and metabolomic changes with the addition of ingredients such as salt, salted fish, and glutinous rice paste [[15], [16], [17]], there is currently a gap in understanding how different varieties of garlic impact the microbial community and metabolomic changes in kimchi.

Therefore, in this study, we investigated the changes in metabolite profiles and microbial communities during the fermentation of kimchi with four different garlic varieties to analyze the effect of each garlic variety on the characteristics of kimchi.

## 2 Materials and methods

### 2.1 Experimental materials

Kimchi cabbage (Hwipalam-glod, Haenam, Korea) and ginger (So-saenggang, Wanju, Korea) were purchased from Western Agricultural and Fishery Market in Gwangju, Korea. Fermented anchovy sauce (Chung Jung Won, DAESANG Corp., Seoul, Korea), sugar (Beksul, CJ Cheil Jedang Corp., Seoul, Korea), red pepper powder (Hansaeng, Seocheon, Korea), and purified salt (Hanju Salt, Ulsan, Korea) were purchased online. Four types of garlic were harvested from Namhae, Changnyeong, Danyang, and Uiseong. A map indicating the cultivation regions of garlic is shown in Fig. S1. All experimental analyses were performed using first-grade analytical reagents obtained from Daejung Co. (Gyeonggi-do, Korea). The water and acetonitrile used for high-performance liquid chromatography (HPLC) were of chromatography grade (Merck, Rahway, NJ, USA).

### 2.2 Kimchi preparation

Kimchi was prepared as previously described [16]. Kimchi cabbage was soaked in 10% (w/v) salt solution for 18 h, washed thrice, drained for 2 h, and cut into 3 × 3 cm pieces. Kimchi seasoning, comprising 23.3% red pepper powder, 2.7% ginger, 16.7% garlic, 6.7% sugar, 20.0% salted anchovy sauce, and 30.7% water, was mixed at specific ratios. The seasoning was added to the salted cabbage at an 85:15 ratio (kimchi cabbage:seasoning) and labeled as CON (kimchi without garlic), NGK (kimchi with garlic from Namhae), CGK (kimchi with garlic from Changnyeong), DGK (kimchi with garlic from Danyang), and UGK (kimchi with garlic from Uiseong). Each kimchi sample (600 g) was individually vacuum-sealed and stored at 4 °C for 42 days. Their characteristics were analyzed every 7 days. Analyses were conducted on five kimchi samples from each group.

### 2.3 Analysis of viable cell count, pH, and titratable acidity

The procedures outlined previously [18] were followed to determine pH, titratable acidity, and the viable cell count of kimchi samples. Kimchi samples were juiced and filtered using gauze after blending. pH measurement was conducted at room temperature (24–26 °C) using a pH meter (TitroLine 5000; SI Analytics GmbH, Mainz, Germany). Total acidity was determined by titration with 0.1 N NaOH until a pH of 8.3 was reached, and titratable acidity was calculated as the percentage of lactic acid produced. Enumeration of colony-forming units (CFUs) for lactic acid bacteria (LAB) and coliforms was carried out using 3 M Petrifilm LAB count plates and coliform count plates (3 M, Saint Paul, MN, USA), respectively, following the manufacturer's instructions. The obtained values were adjusted for relevant dilution factors, and the results were expressed as log CFU/mL.

### 2.4 Microbial community composition analysis

Following the manufacturer's instructions, DNA was extracted from the samples using the PowerSoil DNA isolation kit (MO BIO Laboratories, Carlsbad, CA, USA). Subsequently, concentration and purity were evaluated using a NanoDrop ND-2000 spectrophotometer (Thermo Fisher Scientific, Waltham, MA, USA). PCR amplification targeted the V3–V4 region of the 16S rRNA gene, with MiSeq data classified for each sample using the index sequence as previously described [18]. The PCR protocol involved an initial 2 min denaturation at 95 °C, followed by 30 cycles consisting of 20 s of denaturation at 95 °C, 15 s of annealing at 72 °C, and 1 min of extension at 72 °C, concluding with a final extension lasting 5 min. Subsequent sequencing was conducted on the MiSeq platform (Illumina, San Diego, CA, USA) by Macrogen (Seoul, Korea). Following the removal of errors and chimeric sequences, CD-HIT-OTU analysis was employed to compute species-level operational taxonomic units (OTUs) with a 97% similarity. Using UCLUST in the reference database (SIVA DB), taxonomic assignments were made for representative OTU sequences. The analysis of microbial communities was conducted through the application of Ribosomal Database Project classifiers in QIIME software (v.1.9.2) [19].

### 2.5 Analysis of free sugars

We analyzed free sugars in homogenized kimchi samples. In the initial step, 10 g of each sample was placed in a 50 mL centrifuge tube and adjusted to 50 mL with distilled water. Following this, the solution underwent heating at 85 °C for 25 min, followed by cooling to room temperature (25 °C), and subsequent centrifugation at 3000 rpm for 10 min. One milliliter of the supernatant was filtered through a 0.45 μm nylon membrane filter (Whatman PTFE, Clifton, NJ, USA). For sugar analysis, 6 μL of the filtrate was used. Utilizing HPLC (1260 Infinity; Agilent Technologies, Santa Clara, CA, USA) with refractive index detectors, a carbohydrate column (Asahipak NH2P-50 4E; Shodex, Tokyo, Japan) was employed, maintaining a temperature of 30 °C. The mobile phase, comprising 75% acetonitrile in water, was dispensed at a rate of 1 mL/min. Estimation of free sugar content was performed by employing standard curves for glucose, fructose, sucrose, and mannitol (Sigma-Aldrich, St. Louis, MO, USA).

### 2.6 Gas chromatography coupled with mass spectrometry (GC-MS)-based metabolomic analysis

GC-MS was used for metabolite identification in kimchi samples according to a previously described method [20]. After freeze-drying, samples were treated with O-methoxyamine hydrochloride in pyridine solution (20 mg/mL) for 90 min at 30 °C in the dark. Silylation was performed with *N*-methyl-*N*-trimethylsilyl-trifluoroacetamide containing 1% trimethylchlorosilane, followed by incubation at 37 °C for 30 min. Ribitol (0.5 mg/L) was added as an internal standard. After centrifugation, supernatants were subjected to GC-MS analysis. Quality control samples that were created by pooling equal volumes of each sample were analyzed after every 10 test samples. GC-MS analysis was performed using a Shimadzu QP2020 instrument with a fused silica Rtx-5MS capillary column. The front inlet temperature was 230 °C, and the column temperature ranged from 80 °C to 330 °C. The transfer line and ion source temperatures were 250 °C and 200 °C, respectively. Ionization was achieved with a 70 eV electron beam, and helium flow rate was 1 mL/min. Mass spectra were recorded over 85–500 *m*/*z* at a rate of 20 scans per second. Data analysis was conducted using Shimadzu GC Solutions.

### 2.7 Statistical analysis

The GC-MS data obtained from Shimadzu Postrun Analysis software were processed using MetAlign for peak detection and alignment, resulting in a CSV-format file. AIoutput software was then employed for peak identification and prediction. Multivariate statistical analysis, including principal component analysis (PCA) and partial least squares-discriminant analysis (PLS-DA), was performed using SIMCA-P v15.0 (Umetrics, Umea, Sweden) to confirm metabolic differences between groups. To assess differences in metabolite production during fermentation, statistical analyses was performed using MetaboAnalyst v5.0, which presented results as a heatmap. Data, expressed as mean ± standard deviation from three measurements, underwent one-way analysis of variance and Duncan's multirange tests for statistical significance using XLSTAT basic v19.4 (Addinsoft, New York, NY, USA) (p < 0.05).

## 3 Results and discussion

### 3.1 Chemical properties of garlic cultivars

The four garlic varieties used in this study were cultivated at different locations in the Republic of Korea, such as Namhae, Changnyeong, Danyang, and Uiseong. Garlic from Namhae (NG) and Changnyeong (CG) was classified as a southern ecotype garlic, and garlic from Danyang (DG) and Uiseong (UG) was classified as a northern ecotype garlic. The chemical properties of the four cultivars are listed in Table 1. The moisture content of the garlic varieties was 59.49–65.92; of these varieties, CG had the highest moisture content. In contrast, sugar content was the lowest in CG and the highest in NG at 37.67 brix. Similarly, the levels of both alliin and glucose were the highest in NG, though the alliin content did not show a significant difference between the samples. Notably, the contents of alliin and allicin, compounds associated with the pungency of garlic, differ with the cultivation area and garlic variety [21,22].

**Table 1:** Chemical properties of garlic cultivars.

Components	Namhae (NG)	Changnyeong (CG)	Danyang (DG)	Uiseong (UG)
Appearance				
Water content (%)	59.49 ± 0.61^d^	65.92 ± 0.18^a^	61.96 ± 0.25^b^	60.46 ± 0.27^c^
Sugar content (%, Brix)	37.67 ± 0.58^a^	32.00 ± 0.05^c^	35.67 ± 0.58^b^	35.67 ± 0.58^b^
Alliin (mg/100 g)	971.30 ± 98.40^a^	881.00 ± 8.50^a^	906.60 ± 196.10^a^	884.40 ± 109.40^a^
Glucose (mg/100 g)	208.60 ± 22.60^a^	176.70 ± 52.70^ab^	151.50 ± 8.30^b^	140.80 ± 17.40^b^

^1^ All values are the mean ± SD.

^2^Mean sharing different letters in the same row (a-c) are significantly different (*p* < 0.05).

### 3.2 Changes in fermentation properties

Changes in pH and titratable acidity were closely associated with the LAB involved in kimchi fermentation. The number of bacteria is inversely correlated with pH during fermentation [23]. Indicators such as pH, titratable acidity, and microbial properties involved in kimchi fermentation were monitored weekly for 42 days of fermentation. In particular, titratable acidity and pH are key quality indicators of fermentation and show the ripening stage of kimchi.

**Fig. 1:**
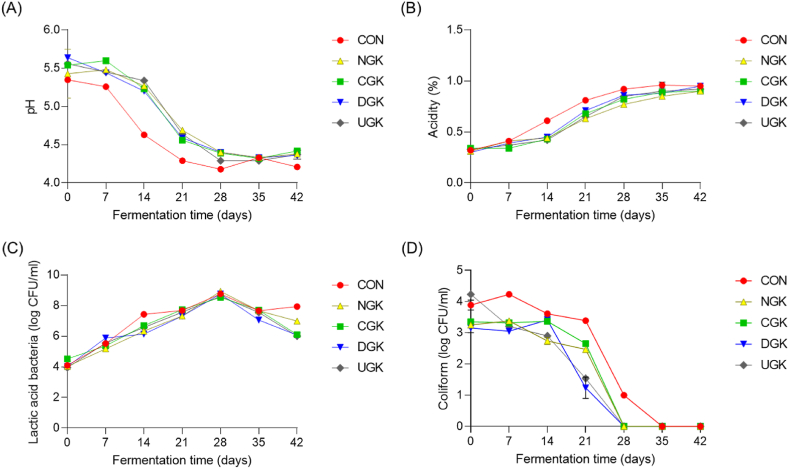
Changes in the pH (A), titratable acidity (B), lactic acid bacteria (LAB) (C), and coliforms (D) of kimchi samples supplemented with different cultivars of garlic at 4 °C during 42 days of fermentation. The LAB and coliforms count is expressed in log CFU/g. CON, kimchi without garlic; NGK, kimchi with garlic from Namhae; CGK, kimchi with garlic from Changnyeong; DGK, kimchi with garlic from Danyang; UGK, kimchi with garlic from Uiseong.

The pH values in the initial stage of fermentation in all kimchi samples were approximately 5.35–5.64 (Fig. 1(A)). The pH value decreased rapidly after 7 days of fermentation, reaching less than 4.5, and then became relatively constant after 28 days. In particular, the pH of the CON, showed a significantly rapid decrease. The titratable acidity of the kimchi samples was 0.32–0.34% in the initial stage of fermentation and increased to 0.90–0.95% at the end of fermentation; the rapid increase in the CON was similar to the decreasing trend observed with pH.

Fig. 1(B) shows the microbial properties of the samples during kimchi fermentation using different types of garlic. In general, lactic acid bacteria tend to increase rapidly during the initial stage of fermentation and then decrease during the end of fermentation of kimchi. Similarly, in this study, the LAB in all samples reached 8 log CFU/g on the 28th day of fermentation, with a slight decrease towards the end of the fermentation period.

While the addition of garlic did not significantly influence the number of LAB in the kimchi, it exhibited a notable impact on coliform levels.

Garlic juice exerts inhibitory effects against bacteria, yeasts, and molds [24]. Similarly, in the present study, coliforms were detected at high levels in kimchi without garlic. In this study, higher levels of coliforms were detected in the kimchi samples without garlic. The antimicrobial properties of garlic are attributed to allicin, a compound produced from alliin by alliinase. Allicin interacts with the thiol group of critical proteins involved in microbial metabolism, thereby disrupting their enzymatic activity [25].

In this study, the pH, acidity, and microbial characteristics were not significantly affected by the garlic variety; however, pH and acidity increased rapidly during fermentation and coliforms were detected at high levels in kimchi without garlic. Therefore, addition of garlic to kimchi was considered to affect its fermentation.

### 3.3 Bacterial community dynamics during kimchi fermentation

**Fig. 2:**
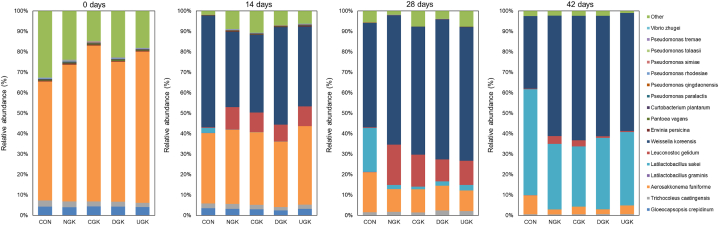
Relative abundance of bacteria at the species level as determined in the SILVA rRNA database. The kimchi samples fermented at 4 °C were collected at 0, 14, 28, and 42 days. ‘Other’ indicates genera showing a percentage of reads <0.5% of the total reads in all the kimchi samples in species-level analyses.

Bacterial species diversity was compared among kimchi samples fermented with different garlic varieties (Fig. 2). *Aerosakkonema funiforme* was the major bacterial strain found in all kimchi samples during fermentation. *Aerosakkonema funiforme* spontaneously grew in the initial stages of fermentation but gradually decreased as fermentation progressed. It is the dominant strain used in kimchi, which is naturally fermented without a starter. The microbial diversity of fermented foods varies depending on the additives used, the fermentation temperature, and duration. By day 7 of fermentation, *Weissella koreensis* was detected in all kimchi samples, but showed the highest proportion in DGK. From the 28th day of fermentation, *W. koreensis* remained the highest in UGK. The population of *W. koreensis* increased rapidly at the beginning of fermentation; thereafter, differences were observed depending on whether garlic was added. In terms of taxonomic composition, *Weissella* exhibited the highest ratio, consistently observed across all fermentation times. Furthermore, as fermentation progressed, the ratio of *Weissella* increased in all groups, with the exception of the CON group (Fig. 2). Following 14 days of fermentation, the proportion of *Latilactobacillus sakei* began to notably increase, reaching particularly high levels in kimchi without garlic (CON). The original bacterial community in the early stages of kimchi fermentation was rapidly replaced by LAB, including *L*. *sakei*, *Leuconostoc gelidum*, and *W*. *koreensis* at the fermentation midpoint. Similar findings have been reported previously [23,26]. The dominant bacteria by kimchi type were also abundantly found in the microbial communities of kimchi cabbage and garlic, which are the raw materials for kimchi. A previous study [7] highlighted that some raw materials (garlic and kimchi cabbage) contain viable fermenting microorganisms as natural microbial groups. These results suggest that the addition of garlic promotes the growth of major kimchi fermentation strains such as *Leuconostoc* and *Weissella*, while inhibiting the growth of strains derived from raw materials. Further, the growth rates of *Leuconostoc* and *Weissela* were affected by the garlic variety used. According to a previous study [5], *Leuconostoc* and *Weissella* possess strong resistance to allicin, a component of garlic. This resistance to allicin during the fermentation of kimchi with garlic is one of the factors that allow these bacterial strains to grow into dominant LAB strains. Overall, these results indicated that the bacterial community profile of kimchi may differ depending on the presence or type of garlic used.

### 3.4 Changes in the free sugar content during kimchi fermentation

**Fig. 3:**
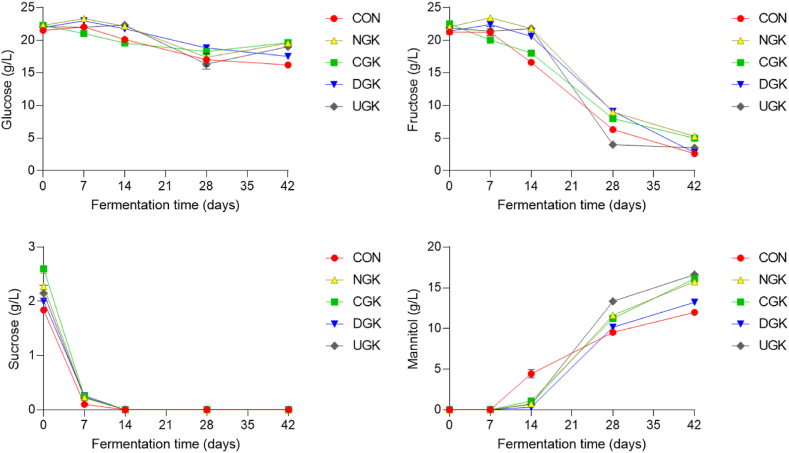
Changes in the free sugar contents of kimchi samples supplemented with different cultivated garlic at 4 °C during 42 days of fermentation.

Kimchi metabolites, including free sugars, amino acids, organic acids, ethanol, and mannitol, are generally closely related to the taste of kimchi. Therefore, these metabolites were analyzed using LC-MS and GC-MS throughout the fermentation process. Free sugar is a carbon source for the bacteria that ferment kimchi and is produced by the degradation of polysaccharides in the cell walls of kimchi cabbage [26,27]. Changes in the free sugar content of kimchi samples containing different garlic varieties are shown in Fig. 3. In this study, the major free sugars responsible for sweetness in kimchi were identified as fructose, glucose, and sucrose. Glucose was found to be the most abundant, followed by fructose and sucrose, consistent with the results of previous reports [9]. The concentration of free sugars in kimchi without garlic (CON) decreased more rapidly than that in kimchi containing garlic. The sucrose content sharply decreased in all kimchi samples until day 7 of fermentation and was undetectable thereafter in all samples. The glucose and fructose contents of kimchi supplemented with NG, which had higher sugar and glucose contents, tended to be relatively higher than those in the other types of kimchi at the beginning of fermentation. Mannitol content was detected after 14 days of fermentation, and at this time, its level in CON increased rapidly compared with that in the other samples. Because the fermentation of kimchi without garlic proceeded quickly, mannitol production was also rapid at the beginning of fermentation in this group. From the 28th day of fermentation, the mannitol concentration was relatively lower than that in the other samples. An early study on kimchi fermentation showed that LAB use fructose as a carbon source and convert it to mannitol, a sugar alcohol that significantly influences kimchi flavor. Mannitol dehydrogenase reduces mannose and fructose to mannitol [28]. In heterofermentation, LAB and other bacteria generate mannitol when fructose is utilized as an electron acceptor [9,29]. A previous study reported that during kimchi fermentation, glucose is released from kimchi cabbage, and most free sugars are converted to mannitol as fermentation progresses [30]. In agreement with these findings, our study observed an increase in the mannitol content of kimchi, concurrent with a decrease in glucose and fructose levels during kimchi fermentation.

### 3.5 Changes in metabolite profiles during kimchi fermentation

The taste and flavor of kimchi are greatly influenced by kimchi metabolites, whose production is significantly shaped by the microbial community during kimchi fermentation [14,26]. Notably, minor ingredients can have a more substantial impact on kimchi metabolites than the main ingredients [11]. In this study, we employed a metabolomics approach with GC-MS to analyze kimchi metabolites, with the aim of investigating the effects of garlic and its varieties on kimchi fermentation.

**Fig. 4:**
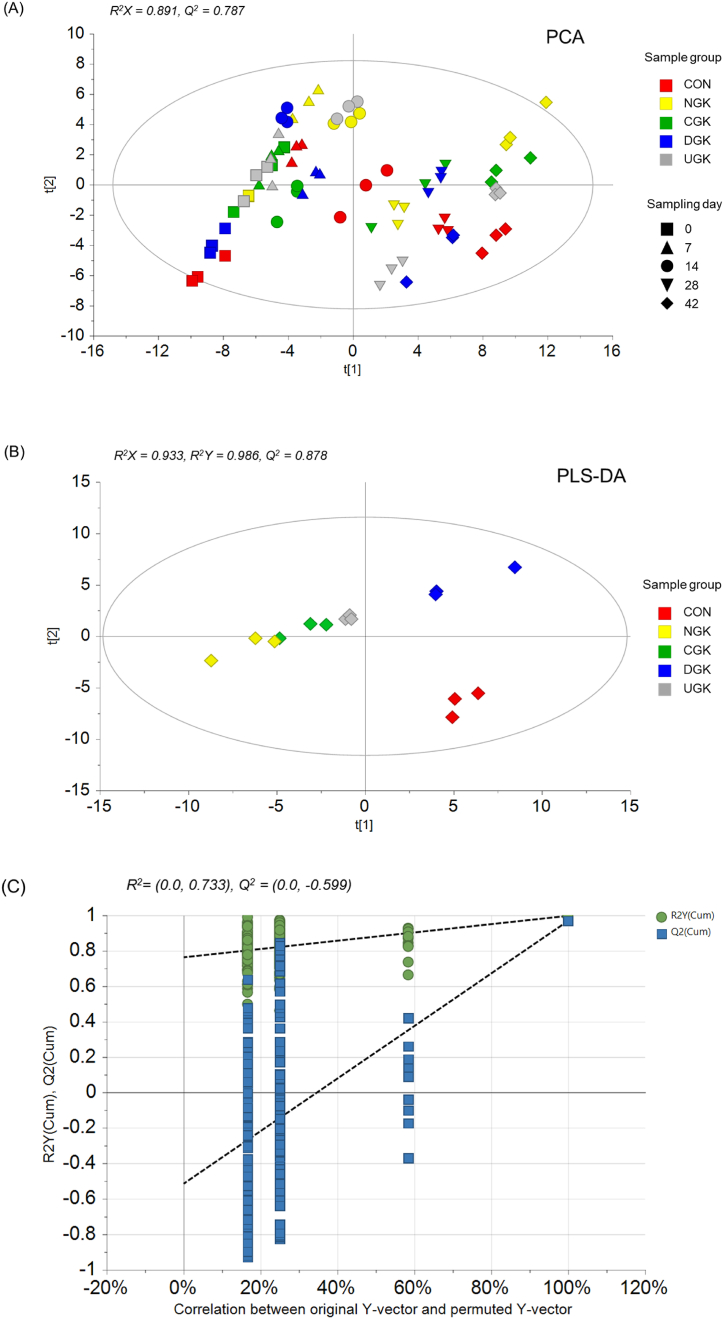
(A) PCA score plots derived from metabolite features of kimchi samples supplemented with different cultivated garlic at 4 °C during 42 days of fermentation. (B) PLS-DA score plots derived from metabolite features of kimchi samples supplemented with different cultivated garlic on 42 day of fermentation. (C) A permutation test was carried out with 200 random permutations in the PLS-DA model.

The PCA score plot showed continuous changes in metabolites during fermentation and a tendency to shift from the left to right over the fermentation period (Fig. 4(A)). The quality parameters of the PCA model were *R*^*2*^*X* = 0.891 and *Q*^*2*^ = 0.787, indicating good suitability and accuracy. In the PCA plot, on days 0, 7, and 14, the metabolites tended to change rapidly during the initial fermentation period, moving along the Y axis. If the sample proceeded slowly from day 28–42 of fermentation, the kimchi metabolites changed slowly. This fermentation metabolite pattern showed a trend similar to that observed in a previous study [16]. In addition, kimchi metabolites were differentiated according to the use of southern ecotype garlic (NG and CG) and northern ecotype garlic (DG and UG).

PLS-DA was used to maximize the separation between samples (Fig. 4(B)). PCA uses only independent variables to obtain a new main component and a regression equation using that main component, whereas PLS considers independent as well as dependent variables to find new variables and uses them to obtain a regression equation [31]. When PLS-DA was applied to kimchi metabolites, a clear difference was observed. When the two components were calculated, the cumulative *R*^*2*^*X*, *R*^*2*^*Y*, and *Q*^*2*^ values were 0.933, 0.986, and 0.878, respectively. A permutation test (200 permutations) was then performed to validate the derived PLS-DA model (Fig. 4(C)). This test demonstrated the suitability and validity of this model by showing that the values of *Q*^*2*^ and *R*^*2*^ were higher than the original values and that the straight intercept between permutations *Q*^*2*^ and original *Q*^*2*^ is negative. In the PLS-DA results, there was a clear difference in the kimchi metabolites at the end of fermentation, depending on whether garlic was added or not and the use of southern and northern ecotype garlic.

**Fig. 5:**
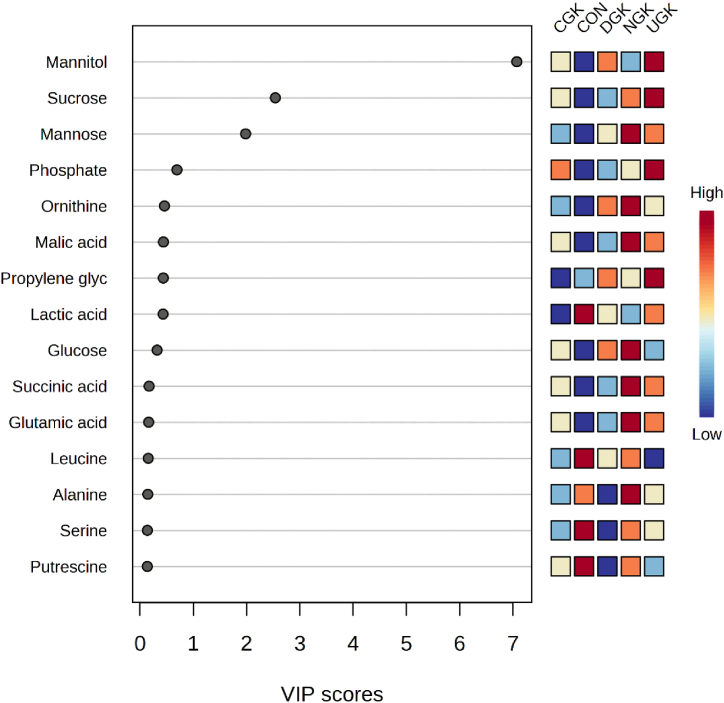
Variable importance in projection (VIP) scores showing major metabolies. The top 15 important metabolites are summarized on the left according to their VIP value. The intensity of the colored boxes indicates the relative metabolite abundance in each group.

Based on the PLS-DA model, we further analyzed the variable importance in projection (VIP) values to determine metabolites with VIP scores greater than 1, representing important differential metabolites. A VIP score greater than 1 indicates a relevant or important variable, whereas a VIP score less than 0.8 indicates an irrelevant or unimportant variable [32]. Analysis of the metabolites produced during fermentation revealed that mannitol, sucrose, mannose, phosphate, ornithine, and malic acid are important metabolites defining the PLS-DA model, which distinguishes the quality of kimchi according to the garlic variety used and whether garlic was added (Fig. 5). The most important metabolite was found to be mannitol, which was significantly affected by the presence or absence of garlic. In the image (Fig. 5) depicting CON (without garlic), the colored boxes for lactic acid, leucine, serine, and putrescine are shown in red, indicating a relatively high abundance of these metabolites. This indicates that these metabolites are likely derived from kimchi without garlic.

**Fig. 6:**
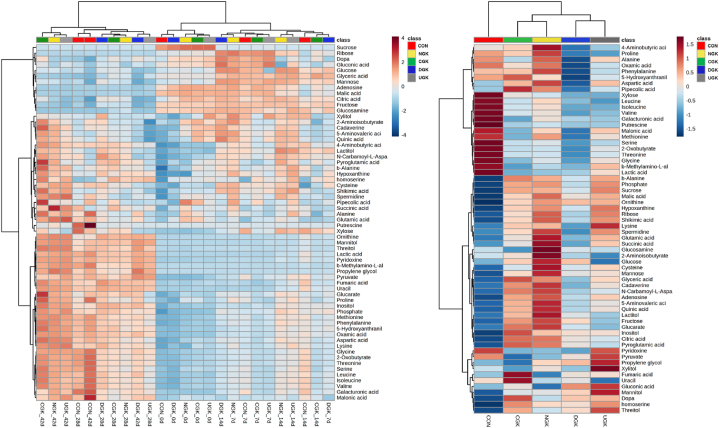
Heatmap showing the metabolite profiles in kimchi samples supplemented with different garlic types during fermentation. The blue and red colors correspond to negative and positive correlation, respectively. The color intensity is proportional to the correlation coefficient. (For interpretation of the references to color in this figure legend, the reader is referred to the Web version of this article.)

In total, 62 metabolites were identified in kimchi samples using GC-MS analysis. The changes in the metabolites identified in each group are shown in Fig. 6 as a heatmap. The levels of some metabolites in kimchi changed markedly during fermentation. Upon comparing the samples on day 42 of fermentation, kimchi without garlic (CON) showed a metabolite profile different from that of kimchi with garlic. In addition, differences in metabolites were clearly observed according to the garlic cultivars used. According to the heatmap, when the metabolites were integrated according to the overall fermentation period, the metabolites of kimchi made with warm ecotype garlic (southern) and of kimchi made with cold ecotype garlic (northern) were divided into respective groups. The variations in the metabolite patterns of kimchi across different regions can be attributed to the differences in the raw materials used. The variations in metabolite patterns are closely associated with the types of microorganisms present, which depend on the specific raw materials used [11]. In addition, even for the same material, the size of the material influences the microbial and metabolite profiles during fermentation [33]. In particular, the differences in these metabolites were caused by the minor ingredients (radish, glutinous rice paste, and seaweed) rather than by the major ingredients. Even if the salt concentration is the same, the type of salt added affects the metabolites of kimchi [18]. In addition, the metabolite profile of kimchi is influenced by the amylose content, even when the same type of starch is added [34]. Therefore, even with the same raw materials, the final metabolite profiles of kimchi could vary depending on the variations in the microorganisms, which is influenced by the condition and size of the raw materials.

The presence and the variety of garlic significantly influenced the metabolites in kimchi, impacting its quality. However one limitation of the study is that it did not analyze the aromatic components. Future research should focus on exploring the effects of alliin on the volatile aromatic components of kimchi, particularly in relation to the different varieties of garlic used.

## 4 Conclusion

In this study, the microbial communities and metabolites generated during kimchi fermentation with and without garlic of four varieties were analyzed and statistically compared to investigate the effects of garlic variety and addition on kimchi fermentation characteristics. The microbial community and metabolites that affect the taste of kimchi were affected by the presence or absence of garlic, and the metabolites produced were found to differ depending on the garlic variety used. Interestingly, there was a clear difference in the metabolites at the end of kimchi fermentation depending on whether southern or northern ecotype garlic was used. Overall, in addition to the main ingredients of kimchi the sub-materials also have a great influence on the taste and aroma of kimchi; therefore, additional research is necessary to understand these effects. In this study, various characteristics of kimchi was demonstrated according to the presence or absence of four kinds of garlic, which is thought to be helpful for standardized kimchi manufacturing.

## Funding statement

This research was supported by a grant from the 10.13039/501100003722World Institute of Kimchi (KE2402-1), funded by the Ministry of Science and ICT, Republic of Korea.

No additional information in available for this paper.

## Data statement

Data will be made available on request.

## CRediT authorship contribution statement

**Yun-Jeong Choi:** Writing - original draft, Formal analysis, Data curation. **Ju-Young Lim:** Investigation, Formal analysis. **Min-Jung Kang:** Formal analysis, Conceptualization. **Ji-Young Choi:** Visualization, Validation. **Ji-Hee Yang:** Methodology, Investigation. **Young Bae Chung:** Validation, Software. **Sung Hee Park:** Validation, Data curation. **Sung Gi Min:** Funding acquisition, Conceptualization. **Mi-Ai Lee:** Writing - review & editing, Project administration, Conceptualization.

## Declaration of competing interest

The authors declare the following financial interests/personal relationships which may be considered as potential competing interests:

## Appendix A Supplementary data

Appendix A. Suplementary material.docx Appendix A. Suplementary material.docx

## References

[1] Kyung K.H., Park K.S., Kim Y.S. (1996). Isolation and characterization of bacteria resistant to the antimicrobial activity of garlic. J. Food Sci..

[2] Seo H., Seong H., Kim G.Y., Jo Y.M., Cheon S.W., Song Y. (2021). Development of anti-inflammatory probiotic *Limosilactobacillus reuteri* EFEL6901 as kimchi starter: in vitro and in vivo evidence. Front. Microbiol..

[3] Ick M.T. (2003). Factors affecting kimchi fermentation. Jpn J Lactic Acid Bact.

[4] Jo Y.M., Seo H., Kim G.Y., Cheon S.W., Kim S.A., Park T.S. (2020). *Lactobacillus pentosus* SMB718 as a probiotic starter producing allyl mercaptan in garlic and onion-enriched fermentation. Food Funct..

[5] Lim S.B., Shin S.Y., Moon J.S., Otgonbayar G.E., Joo W., Lee S.J. (2015). Garlic is a source of major lactic acid bacteria for early-stage fermentation of cabbage kimchi. Food Sci. Biotechnol..

[6] Hong Y., Yang H.S., Chang H.C., Kim H.Y. (2013). Comparison of bacterial community changes in fermenting Kimchi at two different temperatures using a denaturing gradient gel electrophoresis analysis. J. Microbiol. Biotechnol..

[7] Song H.S., Whon T.W., Kim J., Lee S.H., Kim J.Y., Kim Y.B. (2020). Microbial niches in raw ingredients determine microbial community assembly during kimchi fermentation. Food Chem..

[8] Lee S.H., Jung J.Y., Jeon C.O. (2015). Source tracking and succession of Kimchi lactic acid bacteria during fermentation. J. Food Sci..

[9] Jung J.Y., Lee S.H., Lee H.J., Seo H.Y., Park W.S., Jeon C.O. (2012). Effects of *Leuconostoc mesenteroides* starter cultures on microbial communities and metabolites during kimchi fermentation. Int. J. Food Microbiol..

[10] Lee S.H., Whon T.W., Roh S.W., Jeon C.O. (2020). Unraveling microbial fermentation features in kimchi: from classical to meta-omics approaches. Appl. Microbiol. Biotechnol..

[11] Lee D.Y., Park S.H., Park S.E., Kim E.J., Kim H.W., Seo S.H. (2023). Comprehensive elucidation of the terroir of Korean kimchi through the study of recipes, metabolites, microbiota, and sensory characteristics. Food Res. Int..

[12] Park S.-E., Seo S.-H., Kim E.-J., Na C.-S., Son H.-S. (2018). Effects of different fermentation temperatures on metabolites of Kimchi. Food Biosci..

[13] Patra J.K., Das G., Paramithiotis S., Shin H.-S. (2016). Kimchi and other widely consumed traditional fermented foods of Korea: a review. Front. Microbiol..

[14] Seo S.-H., Park S.-E., Kim E.-J., Lee K.-I., Na C.-S., Son H.-S. (2018). A GC-MS based metabolomics approach to determine the effect of salinity on Kimchi. Food Res. Int..

[15] Baek J.H., Kim K.H., Han D.M., Lee S.H., Jeon C.O. (2023). Effects of glutinous rice paste and fish sauce on kimchi fermentation. Lebensm Wiss Technol [Food Sci Technol].

[16] Lee M.-A., Choi Y.-J., Kim Y.-S., Chon S.-Y., Chung Y.B., Park S.-H. (2022). Effects of salt type on the metabolites and microbial community in kimchi fermentation. Heliyon.

[17] Lim J.Y., Choi Y.J., Lee S.Y., Lee M.J., Yang H.I., Kim E.H. (2022). Bacteria composition and metabolites of kimchi as affected by salted shrimp (saeujeot). Int. J. Food Prop..

[18] Lee M.-A., Choi Y.-J., Lee H., Hwang S., Lee H.J., Park S.J. (2021). Influence of salinity on the microbial community composition and metabolite profile in kimchi. Fermentation.

[19] Caporaso J.G., Kuczynski J., Stombaugh J., Bittinger K., Bushman F.D., Costello E.K. (2010). QIIME allows analysis of high-throughput community sequencing data. Nat. Methods.

[20] Seo S.-H., Park S.-E., Kim E.-J., Cho K.-M., Kwon S.J., Son H.-S. (2020). Effect of fungi on metabolite changes in kimchi during fermentation. Molecules.

[21] Nguyen B.T., Hong H.T., O'Hare T.J., Wehr J.B., Menzies N.W., Harper S.M. (2021). A rapid and simplified methodology for the extraction and quantification of allicin in garlic. J. Food Compos. Anal..

[22] Oh H.-L., Kim N.-Y., Sohn C.-W., Ryu B.-R., Yoon J.-H., Kim M.-R. (2012). Analyses of pungency-related factors of field and rice paddy garlic. J Korean Soc Food Sci Nutr.

[23] Jeong S.H., Lee S.H., Jung J.Y., Choi E.J., Jeon C.O. (2013). Microbial succession and metabolite changes during long-term storage of Kimchi. J. Food Sci..

[24] Ji W.D., Jeong M.S., Choi U.K., Choi D.H., Chung Y.G. (1998). Growth inhibition of garlic (*Allium sativum* L.) juice on the microorganisms. Agric. Chem. Biotechnol..

[25] Akullo J.O., Kiage B., Nakimbugwe D., Kinyuru J. (2022). Effect of aqueous and organic solvent extraction on in-vitro antimicrobial activity of two varieties of fresh ginger (*Zingiber officinale*) and garlic (*Allium sativum*). Heliyon.

[26] Park S.-E., Seo S.-H., Kim E.-J., Byun S., Na C.-S., Son H.-S. (2019). Changes of microbial community and metabolite in kimchi inoculated with different microbial community starters. Food Chem..

[27] Ha J.-H., Hawer W.S., Kim Y.-J., Nam Y.-J. (1989). Changes of free sugars in Kimchi during fermentation. Korean J Food Sci Technol.

[28] Wisselink H.W., Weusthuis R.A., Eggink G., Hugenholtz J., Grobben G.J. (2002). Mannitol production by lactic acid bacteria: a review. Int. Dairy J..

[29] Grobben G.J., Peters S.W.P.G., Wisselink H.W., Weusthuis R.A., Hoefnagel M.H.N., Hugenholtz J. (2001). Spontaneous formation of a mannitol-producing variant of *Leuconostoc pseudomesenteroides* grown in the presence of fructose. Appl. Environ. Microbiol..

[30] Yun J.W., Kang S.C., Song S.K. (1996). Mannitol accumulation during fermentation of kimchi. J. Ferment. Bioeng..

[31] Bylesjö M., Rantalainen M., Cloarec O., Nicholson J.K., Holmes E., Trygg J. (2006). OPLS discriminant analysis: combining the strengths of PLS‐DA and SIMCA classification. J. Chemom..

[32] Wold S., Johansson A., Cocchi M. (1993).

[33] Choi H.-W., Park S.-E., Kim E.-J., Seo S.-H., Whon T.W., Son H.-S. (2023). Effects of ingredient size on microbial communities and metabolites of radish kimchi. Food Chem..

[34] Park S.-E., Cho K.-M., Kwon S.J., Kim E.-J., Seo S.-H., Jeong D. (2023). Effects of the addition of starches with different amylose contents on kimchi microbiota and metabolites. Lebensm Wiss Technol [Food Sci Technol].

