# Supplementary material for: Changes in bacterial composition and metabolite profiles during kimchi fermentation with different garlic varieties
Source: Heliyon. 2024 Jan 9;10(2):e24283. doi: 10.1016/j.heliyon.2024.e24283 (PMC10826663; doi:10.1016/j.heliyon.2024.e24283)
Supplement: Appendix A. Suplementary material.docx [file mmc1.docx]

**Supplementary material for:**

**Changes in bacterial composition and metabolite profiles during kimchi fermentation with different garlic varieties**

Yun-Jeong Choi^a^, Ju-Young Lim^a^, Min-Jung Kang^b^, Ji-Young Choi^a^, Ji-Hee Yang^a^, Young Bae Chung^a^, Sung-Hee Park^a^, Sung Gi Min^a^, Mi-Ai Lee^a*^

^a^ *Kimchi Industry Promotion Division, World Institute of Kimchi, Gwangju, 61755, Republic of Korea*

*^b^ Namhae Garlic Research Institute, Namhae, 52430, Republic of Korea*

*Running title: Effects of garlic varieties on kimchi metabolites*

^*^**Corresponding author**: Dr. Mi-Ai Lee

(Practical Technology Research Group, World Institute of Kimchi, Kimchiro 86, Gwangju, 61755, Republic of Korea; Tel: +82 62 610 1733; Fax: +82 62 610 1850; E-mail: leemae@wikim.re.kr)

**Supplementary Figures:**

**Fig. S1.** Indication of cultivated region of garlic.


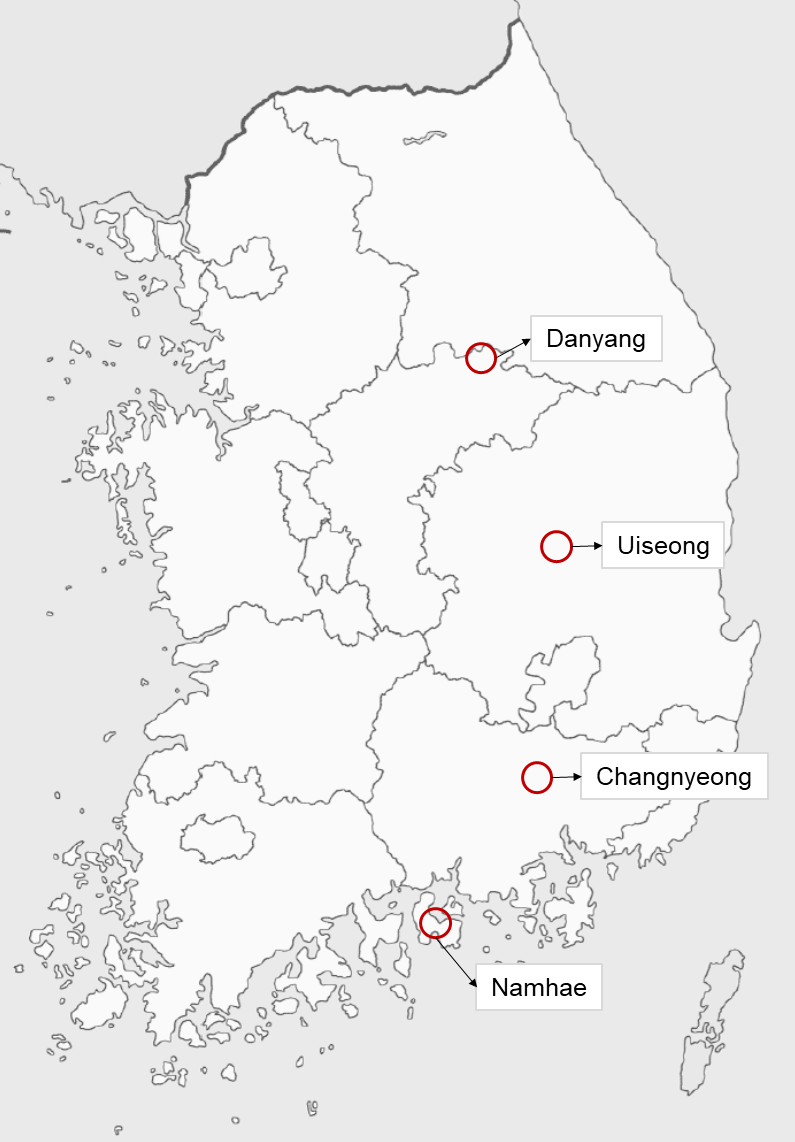


**Fig. S1.** Indication of cultivated region of garlic.
